# Reaching all women, children, and adolescents with essential health interventions by 2030

**DOI:** 10.1136/bmj.l6986

**Published:** 2020-01-27

**Authors:** Ties Boerma, Cesar G Victora, Miriam Lewis Sabin, Paul J Simpson

**Affiliations:** 1 University of Manitoba, Winnipeg, Canada; 2 Countdown to 2030 for Women’s, Children’s and Adolescents’ Health; 3 International Centre for Equity in Health, Federal University of Pelotas, Brazil; 4 Partnership for Maternal, Newborn, and Child Health, Geneva, Switzerland; 5 *The BMJ*, London, UK

## Abstract

A marathon that requires a concerted effort

If the sustainable development goals (SDGs) were a marathon we’d be approaching the 14 km mark after a slow start. There would be more than 28 km still to go, and everyone has to finish the race. We’re entering the final 10 years of the SDGs. Some runners are already falling behind and are at risk of not making the 2030 finishing line.

## Race is on

The precursors to the SDGs, the millennium development goals, created an impetus that led to unprecedented progress in maternal and child health.^1^ Even though reducing inequalities was not explicit in the goals’ 2015 targets, disadvantaged populations often made faster progress than more privileged populations in the goals of improved maternal and child survival and coverage of health interventions mainly because they started from a very low baseline.^2^ ^3^

The progress made should be applauded, but the millennium development goals were a marathon that many participants could not complete. Most of the 2015 targets were not met, and poor-rich and other inequities remained large.

The SDGs’ mantra, which all countries signed up to through the United Nations, is “Leave no one behind.” The 2030 goals target health and wellbeing for all and the UN’s Every Woman Every Child (EWEC) global strategy for women’s, children’s, and adolescents’ health (2016-2030) is the unifying roadmap to achieve that for women, children, and adolescents.^4^

Both the SDGs and the EWEC global strategy emphasise equity and recognise that collecting disaggregated data is the only way of tracking progress and holding governments to account. The need for such data is so important that it became a target in its own right, with SDG target 17.18 calling for data disaggregated by income, gender, age, race, ethnicity, and other important factors.

## What progress has been made?

Is the focus on leaving no woman, no child, and no adolescent behind just rhetoric, or is it leading to measurable change? *The BMJ* and *BMJ Global Health* are launching a new collection of articles that explores the data on health inequalities in an attempt to answer this question, one third of the way into the marathon (www.bmj.com/leaving-no-one-behind).

The collection’s articles are written by Countdown to 2030 for Women’s, Children’s, and Adolescents’ Health, a global collaboration of academics, the World Health Organization, Unicef, and civil society, with support from the Partnership for Maternal, Newborn and Child Health (PMNCH). Three lessons stand out from the collection, which focuses on the coverage of essential health interventions for reproductive, maternal, newborn, child, and adolescent health and nutrition, such as skilled birth attendance, vaccinations, case management of childhood illnesses, improved water supply, and insecticide treated bed nets to prevent malaria.

Firstly, progress is being made in improving coverage of interventions. However, gains tend to slow when national coverage levels get to around 80%, revealing the extra effort needed to reach the most vulnerable people. Furthermore, poor quality of care is a major factor limiting the impact of health services. Many women, children, and adolescents continue to live in dire circumstances, where they are exposed to armed conflict and bear the consequences of the violence and collapse of health services, water supply, and food security.

Secondly, we need better data to know who is being left behind. Tracking progress on SDG indicators currently relies largely on national population surveys with data on household wealth, education, urban or rural residence, and provinces. These data are critical but not enough. Larger sample sizes in national surveys and advancing analysis can tell us more about urban poor people, the poorest of the poor, subnational populations in districts, or ethnic minorities.^5^ Special surveys are also needed for disadvantaged populations such as migrants and people at high risk of HIV.

Routine health facility data are a poorly tapped source of information to track local progress and target interventions. However, this requires major investments to improve the quality, analysis, and use of such data. We should also acknowledge that not everything can be quantified and a more comprehensive understanding of drivers of inequality, including gender bias, is needed through qualitative methods.^6^

Thirdly, the SDGs and EWEC strategy take a comprehensive approach that goes beyond health service provision by including nutrition, violence, early childhood development, and adolescent health and wellbeing. Tackling these challenges requires multisectoral approaches.^7^ Monitoring such a wide array of priority areas—60 indicators in the EWEC strategy—is daunting for all countries and even more so for low income and lower to middle income countries with limited analytical capacity.

## Completing the marathon

A large gap exists between the language of the SDGs and monitoring practices used both globally and by countries. A recent meeting with 10 countries in sub-Saharan Africa on evidence and practices related to monitoring equity for women’s, children’s, and adolescents’ health found that country health plans embraced equity oriented policies towards universal coverage of essential services.^8^ However, although several countries had explicit plans to disaggregate indicators for monitoring progress, systematic monitoring and use of equity data was generally weak.

Leaving no woman, no child, no adolescent behind is a noble goal and an immense challenge. Disaggregated data, reliable transparent statistics, and evidence on health inequalities form the foundation for planning, programme implementation, tracking progress, and accountability. We’re already a third of the way through the SDG race; we must now identify who is being left behind so that we can help everyone complete the marathon by 2030.

## Notes

Provenance and peer review: Commissioned; not externally peer reviewed.
